# MHC class I diversity in chimpanzees and bonobos

**DOI:** 10.1007/s00251-017-0990-x

**Published:** 2017-06-16

**Authors:** Vincent Maibach, Jörg B. Hans, Christina Hvilsom, Tomas Marques-Bonet, Linda Vigilant

**Affiliations:** 1 Department of Primatology, Max Planck Institute for Evolutionary Anthropology, Deutscher Platz 6, 04103 Leipzig, Germany; 2 Copenhagen Zoo, Roskildevej 38, 2000 Frederiksberg, Denmark; 3 Institute of Evolutionary Biology (UPF-CSIC), PRBB, Dr. Aiguader 88, 08003 Barcelona, Catalonia Spain; 4 Catalan Institution of Research and Advanced Studies (ICREA), Passeig de Lluís Companys, 23, 08010 Barcelona, Spain; 5 CNAG-CRG, Centre for Genomic Regulation (CRG), Barcelona Institute of Science and Technology (BIST), Baldiri i Reixac 4, 08028 Barcelona, Spain

**Keywords:** *Pan troglodytes*, *Pan paniscus*, PacBio, Great apes, Next-generation sequencing

## Abstract

Major histocompatibility complex (MHC) class I genes are critically involved in the defense against intracellular pathogens. MHC diversity comparisons among samples of closely related taxa may reveal traces of past or ongoing selective processes. The bonobo and chimpanzee are the closest living evolutionary relatives of humans and last shared a common ancestor some 1 mya. However, little is known concerning MHC class I diversity in bonobos or in central chimpanzees, the most numerous and genetically diverse chimpanzee subspecies. Here, we used a long-read sequencing technology (PacBio) to sequence the classical MHC class I genes *A*, *B*, *C*, and *A-like* in 20 and 30 wild-born bonobos and chimpanzees, respectively, with a main focus on central chimpanzees to assess and compare diversity in those two species. We describe in total 21 and 42 novel coding region sequences for the two species, respectively. In addition, we found evidence for a reduced MHC class I diversity in bonobos as compared to central chimpanzees as well as to western chimpanzees and humans. The reduced bonobo MHC class I diversity may be the result of a selective process in their evolutionary past since their split from chimpanzees.

**Electronic supplementary material:**

The online version of this article (doi:10.1007/s00251-017-0990-x) contains supplementary material, which is available to authorized users.

## Introduction

The major histocompatibility complex (MHC), one of the most gene dense regions within higher vertebrates, plays a critical role in the mammalian immune response (Codner et al. 2012; Geraghty et al. 2002; Kelley et al. 2005). Genes within this complex code for cell surface proteins, which can be grouped into different classes. MHC class I molecules defend against intracellular pathogens by binding intracellular peptides and presenting them to cytotoxic T cells (Sommer 2005). The need to bind a wide range of intracellular peptides selects for a large number of different alleles, making the MHC one of the most polymorphic gene families within mammalian genomes (Geraghty et al. 2002; Kelley et al. 2005; Klein 1987; Kulski et al. 2002; Mayer et al. 1988; Rock et al. 2016; Trowsdale and Parham 2004). In addition, some MHC class I allotypes are involved in the interaction with killer cell immunoglobulin-like receptors (KIR receptors) expressed predominantly on natural killer cells (NK cells) which activate and regulate NK cell responses (Parham 2005; Trowsdale 2001).

Orthologues of the human class I genes *HLA-A*, *-B*, and *-C*, have been found in different great ape species, including chimpanzees (*Pan troglodytes*) and bonobos (*Pan paniscus*) which shared a common ancestor with humans about 7 to 8 million years ago (Adams and Parham 2001; Bontrop 2006; Langergraber et al. 2012; Lawlor et al. 1988; Mayer et al. 1988; Prado-Martinez et al. 2013). Those loci are designated in chimpanzees and bonobos as *Patr-A*, *Patr-B*, *Patr-C*, *Papa-A*, *Papa-B*, and *Papa-C*, respectively (Klein et al. 1990). Because polymorphism in the common ancestor may be retained or lost in speciating lineages, MHC alleles may exhibit trans-species polymorphism, in which some alleles from a certain MHC locus are more closely related to alleles of the orthologous locus in a different species than to alleles from the same locus in the same species (Gyllensten and Erlich 1989; Klein 1987; Klein et al. 1998; Lawlor et al. 1988; Mayer et al. 1988). Furthermore, not all MHC loci are present in all great apes and humans. For example, half of chimpanzees but no bonobos surveyed possess an *A-like* locus, which is nonpolymorphic and orthologous to the orangutan class I *A* and the 5′ part of the *HLA-Y* pseudogene (Adams et al. 2001; Gleimer et al. 2011).

The presence or absence, as well as the relative diversity of alleles at MHC class I loci may give insights into the selective pressures shaping the evolutionary histories of these loci in great apes. For example, researchers proposed that exposure to SIVcpz or another highly pathogenic virus 2–3 million years ago may be responsible for the low diversity found in a sample of chimpanzee MHC class I loci *A*, *B* and *C* intron 2 sequences, as well as the low variation observed in a survey using microsatellite loci across the chimpanzee MHC class I and II regions (de Groot and Bontrop 2013; de Groot et al. 2008, 2002). More direct evidence for the influence of SIVcpz was inferred from a study of MHC class I *B* allelic variation in a wild population of 125 eastern chimpanzees (*P.t. schweinfurthii*) including three communities varying in SIVcpz prevalence (Wroblewski et al. 2015). The community with the highest prevalence of SIVcpz had significantly higher frequencies of particular *B* alleles, including an allele with high similarity to the HLA-B*57 allele associated in humans with less viral load and delayed progression of HIV infection (Wroblewski et al. 2015).

It is, however, important to note that sampling at nearly 90 field sites revealed SIV infection in only two of the four chimpanzee subspecies, namely the central (*P.t. troglodytes*) and eastern (*P.t. schweinfurthii*) subspecies, but not the western (*P.t. verus*) or Nigeria-Cameroonian (*P.t. ellioti*) subspecies (Heuverswyn et al. 2007; Keele et al. 2006; Rudicell et al. 2010; Santiago et al. 2003; Santiago et al. 2002; Sharp and Hahn 2011; Worobey et al. 2004). This could mean that SIVcpz, or other pathogens, may still be in the process of differentially shaping the MHC diversity of chimpanzee subspecies today. Comparisons are difficult because MHC class I diversity has largely been characterized from representatives of just the western or eastern subspecies. After over 20 years of MHC class I studies in chimpanzees, the number of individuals characterized from the different subspecies greatly varies, with data available from approximately 85 western chimpanzees and 130 eastern chimpanzees but only some 15 central chimpanzees, indicating that central chimpanzees are clearly underrepresented in the description of MHC class I diversity. Furthermore, it is interesting to note that individuals from the western and eastern chimpanzee subspecies apparently have no common alleles at the *B* locus, suggesting that there may indeed be pronounced differences in MHC variation between the subspecies (Wroblewski et al. 2015). Chimpanzee subspecies also differ in demographic histories. Comparisons revealed highest genetic diversity and effective population size in the central chimpanzee (Becquet et al. 2007; Fischer et al. 2006, 2004; Hey 2010; Prado-Martinez et al. 2013; Wegmann and Excoffier 2010). Central chimpanzees also showed signs of population growth, whereas western chimpanzees and eastern chimpanzees showed signs of a decrease in effective population size (Caswell et al. 2008; de Manuel et al. 2016; Fischer et al. 2011, 2004). The different demographic histories of the subspecies which may have influenced also the shaping of the MHC diversity in the past, the different pathogenic pressures of the subspecies and the underrepresentation of central chimpanzees in the description of MHC class I diversity all present a compelling argument to analyze MHC diversity specifically in central chimpanzees. This would contribute to the understanding of the whole diversity in this species and could eventually reveal signs of selection in these important loci in central chimpanzees in particular.

The closest relative of the chimpanzee is the bonobo, and these species last shared a common ancestor between 1 and 2.6 mya (Langergraber et al. 2012; Prufer et al. 2012). As with the different chimpanzee subspecies, chimpanzees and bonobos may have experienced different selective pressures influencing the MHC diversity in those two species, and it is notable that SIVcpz has not been detected in bonobos (Li et al. 2012; Sharp and Hahn 2011). Although MHC variation has been characterized in far more than 100 chimpanzees, assessment of bonobo MHC class I diversity has been limited to a few individuals in various studies adding up to four, four, and nine individuals for the *A*, *B*, and *C* class I loci, respectively (Cooper et al. 1998; Lawlor et al. 1995; Martinez-Laso et al. 2006; McAdam et al. 1994; McAdam et al. 1995). Characterization of MHC variation in additional representatives of the bonobo as well as the chimpanzee will allow assessment of diversity within as well as across these species and may contribute to understanding the evolutionary history of the human MHC.

Although studies of great ape genome diversity are increasingly common (Cagan et al. 2016; de Manuel et al. 2016; Prado-Martinez et al. 2013; Scally et al. 2012) MHC variation is more difficult to assess due to the highly repetitive nature of the region and the short read lengths of most next-generation sequencing platforms, which both make the subsequent assembly and unambiguous reconstruction of the alleles challenging. However, the PacBio RS II system features sequencing of longer molecules and has been used to sequence complete MHC alleles, making this platform a practical solution for MHC typing of humans and non-human primates (Mayor et al. 2015; Westbrook et al. 2015). Here, we used the PacBio RS II system to sequence the MHC class I *A*, *B*, *C*, and *A-like* loci in a sample of wild-born bonobos and chimpanzees, including three different chimpanzee subspecies. We use these data and published information from humans in order to describe the MHC class I diversity in bonobos and elucidate comparative levels of diversity between those species.

## Materials and methods

### Samples

We analyzed 21 bonobo (*P. paniscus*) and 30 chimpanzee (*P. troglodytes*) individuals, including representatives of three of the four chimpanzee subspecies (20 *P.t. troglodytes*, 4 *P.t. verus*, and 6 *P.t. schweinfurthii*). Blood samples of 20 bonobos (*P. paniscus*) and 20 central chimpanzees (*P.t. troglodytes*) were collected in African sanctuaries (Lola ya Bonobo sanctuary Kinshasa, Democratic Republic of Congo, and Tchimpounga sanctuary, Jane Goodall Institute, Republic of Congo, respectively) in 2007 and 2008 by Michel Halbwax and Anne Fischer (Fischer et al. 2011). Individuals were considered unrelated as described in Fischer et al. (2011). We also included one female bonobo (Ulindi, blood sample collected in the Leipzig Zoo in 2011), who was the source for the bonobo genome project (Prufer et al. 2012). Details regarding the samples are listed (Table S1). Genomic DNAs were extracted using the Gentra Puregene Blood Kit (QIAGEN) or QIAamp DNA Blood Mini Kit (QIAGEN). All individuals in this study were either born in the wild or have wild-born parents (Ulindi).

### DNA amplification

We amplified full-length PCR products for each of the four loci (*A*, *B*, *C*, and *A-like*) separately using a primer set for each locus designed to span all exons and introns (see Table S2). Primers for the *B* and *C* loci were taken from Hans et al. (2017). Products for *A*, *B*, *C*, and *A-like* targets were 3412–3440 bp, 3019–3049 bp, 3067–3080 bp long and 3156 bp, respectively. PCRs were set up in a 50 μl volume containing 5× Crimson LongAmp Taq Reaction Buffer (New England BioLabs, Ipswich, Massachusetts, USA), 2.5 mM of each dNTP, 10 μM of each forward and reverse primer, 1 U of Crimson LongAmp Taq DNA Polymerase (New England BioLabs, Ipswich, Massachusetts, USA) and 50 ng DNA. Amplifications started with an initial denaturation step of 2 min at 94 °C, followed by 40 cycles of 20 s at 94 °C, 60 s at 68 °C, 8 min at 68 °C and finished by a final extension step of 15 min at 68 °C. We conducted for all individuals two independent PCR replicates for each locus and verified successful amplification with gel electrophoresis. Amplicons were purified with AGENCOURT AMPure XP PCR purification (Agencourt Bioscience Corporation, Beverly, Massachusetts, USA) following suppliers instructions, except that we changed the ratio between PCR product and AMPure XP Magenetic Particle Solution to 1:1. DNA concentrations of purified amplicons were measured using NanoDrop 1000 (Thermo Fisher Scientific, Waltham, Massachusetts, USA).

Not all chimpanzees have the *A-like* locus, and it has not been detected in bonobos (Adams et al. 2001). To test whether our primers for the *A-like* locus (Table S2) were able to identify all individuals having the *A-like* ^*+*^ haplotype and confirm the lack of this locus in bonobos, for all samples, we performed an additional PCR based on a *Patr-A-like* typing system described in Adams et al. (2001). PCR was done using AmpliTaq Gold with GeneAmp Kit (Thermo Fisher Scientific, Waltham, Massachusetts, USA) and contained the following reagents in a total of 20 μl: 10× GeneAmp PCR Buffer II, 25 mM MgCl_2_, 2.5 mM dNTPs, 10 μM primer AL4S, 10 μM primer AL5R, 0.5 units AmpliTaq Gold DNA Polymerase, and 50 ng DNA. After 9 min at 95 °C, we used 30 cycles with 30 s at 94 °C, 30 s at 58 °C, 1 min at 72 °C and followed by a final elongation of 10 min at 72 °C to amplify the 423-bp product. Successful amplification was checked with gel electrophoresis.

### Pacific Bioscience library preparation

We prepared SMRTbell libraries for PacBio RS II sequencing following the manufacturer’s protocol for Multiplex SMRT Sequencing with PacBio Barcoded Adapters (Pacific Biosciences, Menlo Park, CA, USA). Prior to library preparation, amplicons of the three (*A*, *B*, *C*) or four (*A*, *B*, *C*, *A-like*) different loci, respectively, were pooled in equimolar amounts for each replicate of each individual. Each library contained ten such pooled samples with a DNA concentration of 100 ng per sample. Library preparations were performed according to supplier’s instructions except that after the damage repair step, we again added 1 μl of DNA Damage Repair Mix (Pacific Biosciences, Menlo Park, CA, USA) to each library and incubated the mixture for 30 min at 37 °C. This second damage repair step improved the sequencing by preventing early terminations in the sequencing process due to damages in the DNA template. Sequencing was performed on a PacBio RS II system (Pacific Biosciences, Menlo Park, CA, USA) using P6-C4 chemistry and 240 min movies.

### Sequence data processing

We used the Long Amplicon Analysis tool based on the Quiver framework within the Pacific Bioscience SMRT Analysis System version 2.3.0.140936 (Pacific Biosciences, Menlo Park, CA, USA) to generate high-quality consensus sequences for each amplicon. The amplicons were demultiplexed and assigned to distinct loci according to their primer sequences. For each individual we compared results from the two replicates of each amplicon of each locus. In all but three individuals (Malou_L, Lodja, and Cindy), the replicate sequences were identical. For these three individuals, we found at one locus each (*Papa-C*, *Papa-C*, and *Patr-A*, respectively), two alleles in one replicate in contrast to only one allele in the other replicate. Furthermore, the solitary allele showed a deletion within the coding region at the position which distinguished the other two alleles, suggesting that the algorithm sometimes had difficulties in distinguishing two very similar alleles. For identification of the correct alleles for these three individuals, we used the minor variant protocol of the SMRT Analysis (Pacific Biosciences, Menlo Park, CA, USA), which aligns sequences to a reference and calls variants within that alignment. For each individual, we used in both replicates all three alleles as a reference separately, resulting in six analysis runs per individual. Furthermore, each alignment generated by the minor variant protocol was checked by eye, using samtools version 0.1.19 (Li et al. 2009) for visualizing the data. The minor variant analysis (data not shown) and the visualization of the alignment confirmed that the two alleles found within one replicate are the true alleles of each individual. The fact that those alleles were already described in the literature or we found them in other individuals within our data set and alleles with the deletion had premature stop codons also supported this finding. The spurious alleles with the deletion were discarded. We sent all full-length MHC sequences to the IPD-MHC database for official designation of novel identified alleles (Groot et al. 2012).

### Checking for artifacts

To ensure that we had enough template for the sequencing process, we used 40 PCR cycles (see the “DNA amplification” section). Although within the range of the manufacturer’s recommendation, this is a relatively large number of cycles and may promote the formation of chimeric sequences (Kanagawa 2003; Lenz and Becker 2008). We undertook three measures to reduce the possibility of and check for potential chimeric sequences. First, because chimeric sequences arise mainly during late PCR cycles from incomplete PCR products of earlier cycles, we used an excessively long elongation time of 8 min (see the “DNA amplification” section) to ensure complete elongation of the products and reduce incomplete PCR products to a minimum (Kanagawa 2003; Lenz and Becker 2008). Second, we did every PCR of every locus and of every individual in duplicate, as it is unlikely that identical chimeric sequences would occur in both PCR replicates. Furthermore, any PCR artifacts should have a lower copy number than the target amplicon, which should result in a lower number of reads after the sequencing process. We did not recognize any discrepancies between the two replicates in terms of different sequences or number of reads, except those detailed above (see the “Sequence data processing” section). Lastly, the Long Amplicon Analysis tool of the Pacific Bioscience SMRT Analysis System used for generating the consensus sequences incorporates a chimera detection step using the UCHIME algorithm, which marks all potential chimera sequences and removes them from the further analysis (Edgar et al. 2011; Pacific Biosciences 2014). In summary, using those steps above, we are confident that the sequences reported here do not include chimera sequences.

### Data analysis

We used MEGA 6.06 to construct maximum likelihood trees including all different bonobo and chimpanzee coding region sequences from our data set (Tamura et al. 2013). We added all bonobo and chimpanzee sequences from the literature which were not represented by our own sequences. For human references we included sequences for the *A*, *B*, and *C* loci from Yoruba individuals from the HapMap project (The International HapMap C 2005). In total, the HapMap project sampled 30 triplets of Yoruba individuals (parents and one adult offspring). We took representative alleles from all parents (60 individuals) for the construction of phylogenetic trees. For the construction, the best substitution model was selected using MEGA 6.06. We used a Tamura 3-parameter model with discrete gamma distribution with invariable sites (G + I) for all trees.

We calculated nucleotide diversity ∏ at each locus for 20 bonobos, after excluding Ulindi to have matched sample size to central chimpanzees and 20 central chimpanzees, including both alleles for each individual, with R (version 3.1.3) and the R package “pegas” (version 0.9). We estimated ∏ for introns and exons separately. Additionally, we included data from 20 published western chimpanzees (Adams et al. 2000) and estimated the nucleotide diversity. For comparison with humans, we reduced the Yoruba HapMap dataset to 30 individuals (every first individual of triplet) and chose randomly 20 individuals from this reduced dataset.

We conducted a permutation test (Adams and Anthony 1996) in R (version 3.1.3) for pairwise comparisons of diversity. We pooled all individuals of the two taxa being compared, resampled individuals (to account for non-independence of alleles in the same individual) independently of their taxa origin to compose two groups of the same size as the original two taxa, and calculated the nucleotide diversity of each new group and the absolute difference in nucleotide diversity between the two groups. The absolute difference in nucleotide diversity between the two groups of each permutation was compared to the absolute difference in nucleotide diversity of the original two taxon groups. The *p* value was calculated as the proportion of absolute differences of every permutation greater than or equal to the difference of the two original species (based on 10,000 permutations into which we included the original data as one permutation). We accounted for multiple testing and used a Bonferroni correction. After correction, *p* values below or equal to 0.002 were defined as being significant.

## Results

In our sample of 30 chimpanzees and 21 bonobos, we found 148 different full-length MHC class I alleles (Genbank accession numbers: KY613033–KY613180), including 42 and 21 novel coding region sequences in chimpanzees and bonobos, respectively. From our chimpanzee samples, we obtained 30, 41, 29, and 11 different full-length alleles for the *Patr-A*, *-B*, *-C*, and *-A-like* loci, respectively (Table 1). We found 25, 39, 26, and 3 different coding region sequences for the four loci, respectively, including 8 *A*, 20 *B*, 13 *C*, and 1 *A-like* sequences, which have not been described previously (Table 1). Of these novel coding region sequences Patr-B*20:01:02, Patr-B*23:01:02, Patr-C*03:02:02, and Patr-C*11:01:02 each had one synonymous substitution to known chimpanzee MHC coding region sequences. The translation of all coding region sequences from our chimpanzee sample resulted in 25, 39, 25, and 3 different and functional protein sequences for the *A*, *B*, *C*, and *A-like* loci. The results of the *A-like* screening were in concordance with the successful amplification and sequencing of a full-length *A-like* sequence for individuals carrying the *A-like* ^*+*^ haplotype.

**Table 1:** Chimpanzee MHC class I alleles obtained from 30 individuals. Novel alleles are written in bold. Novel alleles include changes either in the introns or in the coding region sequence or in the protein sequence (allotype). Novel protein sequences (allotypes) are indicated with an asterisk. The three different chimpanzee subspecies *P.t. schweinfurthii* (P.t.s*.*), *P.t. verus* (P.t.v*.*), and *P.t. troglodytes* (P.t.t.) are indicated

	*Patr-A*		*Patr-B*		*Patr-C*		*Patr-AL*		Subspecies
Bihati	A*15:01:01:01	A*08:01:01:01	B*07:04*	B*23:06*	C*07:02*	C*09:05	AL*01:01:01:04		P.t.s.
Cleo	A*22:01	A*08:01:01:01	B*38:02*	B*23:04	C*02:05	C*09:05	AL*01:01:01:06		P.t.s.
Diana	A*27:01*	A*15:01:01:01	B*22:04	B*22:05	C*17:02*	C*03:07*	AL*01:01:01:08		P.t.s.
Maya	A*15:01:01:02	A*13:01:01:02	B*23:05	B*33:01:01:02	C*09:02:01:01	C*09:05	AL*01:01:01:08		P.t.s.
Tongo	A*15:01:01:01	A*08:01:01:01	B*30:02*	B*22:03	C*03:05*	C*09:01:01:04	AL*01:01:01:04		P.t.s.
Trixie	A*15:02		B*06:03	B*07:05*	C*11:01:02	C*09:02:01:02	AL*01:02		P.t.s.
Alice	A*09:01		B*36:01	B*01:01	C*15:01	C*04:01	AL*01:01:01:01		P.t.v.
Berta	A*03:02	A*09:01	B*20:01:02	B*01:01	C*12:01	C*04:01	AL*01:01:01:01		P.t.v.
Cindy	A*05:01	A*04:04	B*13:01		C*03:01		AL*01:01:01:01		P.t.v.
Linda	A*06:02	A*03:01	B*04:02	B*24:01	C*16:01	C*09:01:01:04	*–*		P.t.v.
Agnagui	A*25:01:01:01*	A*17:01:01:01	B*11:03*	B*12:03*	C*03:02:02:01	C*15:02*	*–*		P.t.t.
Bailele	A*26:01*	A*17:04*	B*06:03	B*18:01	C*11:01:02	C*09:01:01:03	AL*01:01:01:02		P.t.t.
Bayokele	A*10:01	A*18:01	B*11:03*	B*22:01	C*15:02*	C*09:02:01:01	AL*01:01:01:03		P.t.t.
Bimangou	A*13:01:01:01	A*17:04*	B*07:02	B*11:04*	C*07:03*	C*09:01:01:04	AL*01:01:01:05		P.t.t.
Botsomi	A*18:01		B*07:03	B*35:02*	C*07:03*	C*03:02:02:02	*–*		P.t.t.
Casimir	A*27:01*	A*08:01:01:01	B*22:04	B*30:02*	C*03:07*	C*09:01:01:04	AL*01:01:01:06		P.t.t.
Castro	A*13:01:01:01	A*25:01:01:02*	B*22:07*	B*06:03	C*11:01:02	C*04:02*	AL*01:01:01:05		P.t.t.
Chinoc	A*15:01:01:01		B*22:06*		C*13:03		AL*01:01:01:07		P.t.t.
Clara_T	A*16:01	A*17:01:01:02	B*40:01:01:01*	B*22:06*	C*03:02:02:02	C*03:02:02:01	AL*01:01:01:06	AL*01:01:01:05	P.t.t.
Dzeke	A*25:01:01:01*	A*12:01	B*11:03*	B*23:01:02	C*15:02*	C*10:02*	*–*		P.t.t.
Elikia	A*17:01:01:01	A*17:04*	B*11:03*	B*18:01	C*15:02*	C*09:01:01:04	*–*		P.t.t.
Fan Tuek	A*03:06*	A*13:01:01:01	B*22:06*	B*11:02	C*03:02:01	C*13:03	AL*01:04*		P.t.t.
Gao	A*03:05*	A*15:01:01:01	B*19:03*	B*40:01:01:01*	C*03:02:02:02	C*13:03	AL*01:01:01:09	AL*01:01:01:07	P.t.t.
Golfi	A*26:01*	A*18:01	B*40:01:01:02	B*40:02*	C*03:02:02:01		AL*01:01:01:02		P.t.t.
Grand Maitre	A*03:07*	A*15:03*	B*23:07*	B*11:03*	C*15:02*	C*17:01*	AL*01:02		P.t.t.
Imphondo	A*16:01	A*03:07	B*35:02	B*21:02*	C*09:04	C*13:04*	AL*01:01:01:05		P.t.t.
Loufoumbou	A*17:01:01:01	A*08:02	B*19:04*	B*21:01	C*09:04	C*13:01	AL*01:01:01:05		P.t.t.
Lufino	A*08:01:01:02	A*18:01	B*23:02	B*33:01:01:01	C*03:02:02:01	C*13:03	AL*01:01:01:06		P.t.t.
Marcelle	A*18:01		B*23:02	B*06:02	C*03:06*	C*03:02:02:01	*–*		P.t.t.
Moka	A*18:01		B*33:01:01:01	B*11:03*	C*15:02*	C*13:03	*–*		P.t.t.
No. of different alleles	30		41		29		11		
No. of different coding region sequences	25		39		26		3		
No. of different allotypes	25		39		25		3		
No. of novel alleles	13		22		17		9		
No. of novel coding region sequences	8		20		13		1		
No. of novel allotypes	8		18		11		1		

Our bonobo sample revealed 13, 13, and 11 different full-length alleles for the *Papa-A*, *-B*, and *-C* loci, respectively (Table 2). We found no *A-like* allele or had a positive *A-like* screening result within our bonobo samples, which is consistent with the presence of the *A-like* locus only in chimpanzees. After removing introns from the full-length alleles, we obtained 11, 13, and 10 different coding region sequences for the three loci *A*, *B*, and *C*, respectively, and of these 11, 3, and 7 were not previously described. Each coding region sequence from our bonobo sample predicted a different and functional protein sequence. The number of different full-length alleles, coding region sequences, and protein sequences for each of three loci (*A*, *B*, and *C*) was higher in the chimpanzee sample compared to the bonobo sample, even if considering only the 20 central chimpanzees (Table 3).

**Table 2:** Bonobo MHC class I alleles obtained from 21 individuals. Novel alleles are written in bold. Novel alleles include changes either in the introns or in the coding region sequence or in the protein sequence (allotype). Novel protein sequences (allotypes) are indicated with an asterisk

	*Papa-A*		*Papa-B*		*Papa-C*	
Api	A*03:06*	A*03:03:01:02*	B*01:03*	B*07:01:02	C*04:01	C*03:02
Bandundu	A*05:03*		B*07:01:02		C*03:02	
Bili_L	A*06:03:01:01*	A*08:03*	B*02:02	B*15:01	C*03:01:01:03	C*06:01:01:02*
Boende	A*03:06*		B*15:01		C*06:01:01:02*	
Bolobo	A*05:03*		B*07:01:02		C*03:02	
Fizi	A*03:02*	A*08:03*	B*09:01	B*19:02	C*04:02*	C*03:02
Isiro	A*05:03*	A*06:03:01:01*	B*02:02	B*07:01:02	C*03:01:01:03	C*03:02
Keza	A*09:02*	A*08:02*	B*01:03*	B*17:01	C*04:01	C*05:02*
Kikwit	A*06:03:01:02*	A*05:03*	B*09:01	B*07:01:02	C*04:02*	C*03:02
Kisantu	A*05:03*	A*08:03*	B*07:01:02		C*03:02	
Kubulu	A*03:06*	A*06:03:01:03*	B*15:01	B*19:01	C*06:01:01:02*	C*05:01*
Likasi	A*06:03:01:01*		B*02:02		C*03:01:01:03	
Lipopo	A*03:05*		B*19:02	B*16:01	C*03:03:01:01*	C*05:03*
Lodja	A*08:03*	A*03:02*	B*07:01:02	B*19:02	C*03:02	C*03:03:01:01*
Lomami	A*08:03*	A*06:03:01:03*	B*19:02	B*14:01	C*03:03:01:01*	C*04:03*
Malou_L	A*08:03*	A*04:02*	B*19:02	B*07:01:02	C*03:02	C*03:03:01:01*
Matadi	A*08:03*	A*06:03:01:02*	B*15:02*	B*07:01:02	C*03:02	C*06:01:01:02*
Max	A*05:03*	A*06:03:01:01*	B*02:02	B*15:01	C*03:01:01:03	C*06:01:01:02*
Semwendwa	A*06:02	A*04:02*	B*01:02*	B*08:01:01	C*04:02*	C*03:03:01:02
Tshilomba	A*03:02*	A*03:06*	B*14:01	B*07:01:02	C*03:02	C*04:03*
Ulindi	A*05:03*	A*03:02*	B*07:01:02		C*03:02	
No. of different alleles	13		13		11	
No. of different coding region sequences	11		13		10	
No. of different allotypes	11		13		10	
No. of novel alleles	13		3		8	
No. of novel coding region sequences	11		3		7	
No. of novel allotypes	11		3		7

**Table 3:** Comparison of the number of full-length alleles, coding region sequences, and protein sequences among bonobos (*n* = 21), chimpanzees including all three subspecies (*n* = 30), and chimpanzees only from the central subspecies (*n* = 20)

	*A*			*B*			*C*			*A-like*		
Species	Bonobo	Chimpanzee (3 ssp.)	Chimpanzee (central)	Bonobo	Chimpanzee (3 ssp.)	Chimpanzee (central)	Bonobo	Chimpanzee (3 ssp.)	Chimpanzee (central)	Bonobo	Chimpanzee (3 ssp.)	Chimpanzee (central)
No. full-length alleles	13	30	20	13	41	26	11	29	20	–	11	8
No. coding region sequences	11	25	18	13	39	25	10	26	17	–	3	3
No. protein sequences	11	25	18	13	39	25	10	25	16	–	3	3

**Fig. 1:**
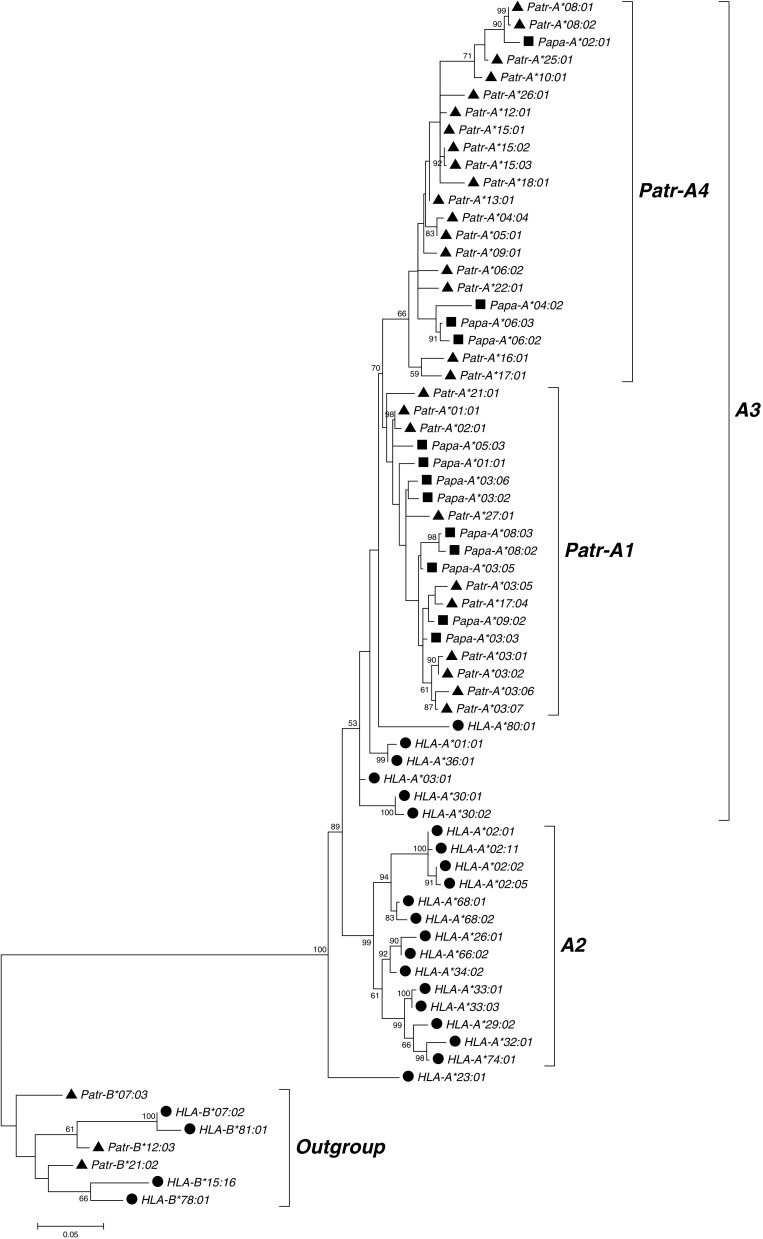
Molecular phylogenetic tree of MHC class I *A* sequences reconstructed using the maximum likelihood method based on the Tamura 3-parameter model. The model used a discrete gamma distribution allowing evolutionary rate differences among sites. The tree with the highest log likelihood (−5799.1552) is shown. Confidence in the branching patterns is indicated by bootstrap values and only relevant values (≥50) are shown. The scale indicates the number of substitutions per site. The tree was rooted with an outgroup consisting of the indicated *MHC-B* sequences. The three species humans, chimpanzees, and bonobos are represented by *circles*, *triangles*, and *squares*, respectively, in front of the sequence names. Different phylogenetic groups are defined on the right side of the tree, showing the two *MHC-A* lineages *A2* and *A3* and the two chimpanzee- and bonobo-specific groups within the *A3* linage *Patr-A1* and *Patr-A4*

**Fig. 2:**
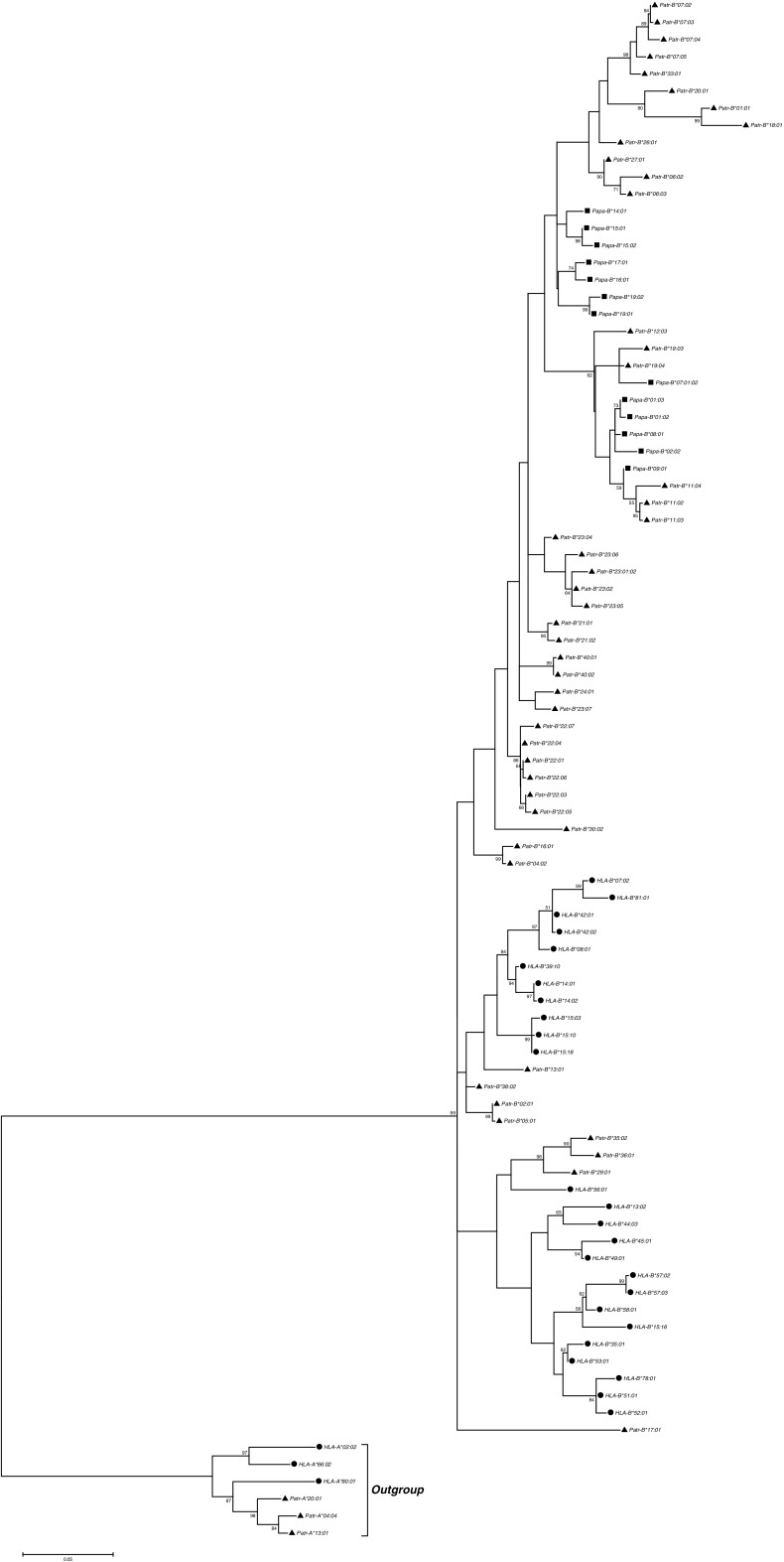
Molecular phylogenetic tree of MHC class I *B* sequences reconstructed using the maximum likelihood method based on the Tamura 3-parameter model. The model used a discrete gamma distribution allowing evolutionary rate differences among sites. The tree with the highest log likelihood (−7151.1817) is shown. Confidence in the branching patterns is indicated by bootstrap values and only relevant values (≥50) are shown. The scale indicates the number of substitutions per site. The tree was rooted with an outgroup consisting of the indicated *MHC-A* sequences. The three species humans, chimpanzees, and bonobos are represented by *circles*, *triangles*, and *squares*, respectively, in front of the sequence names

**Fig. 3:**
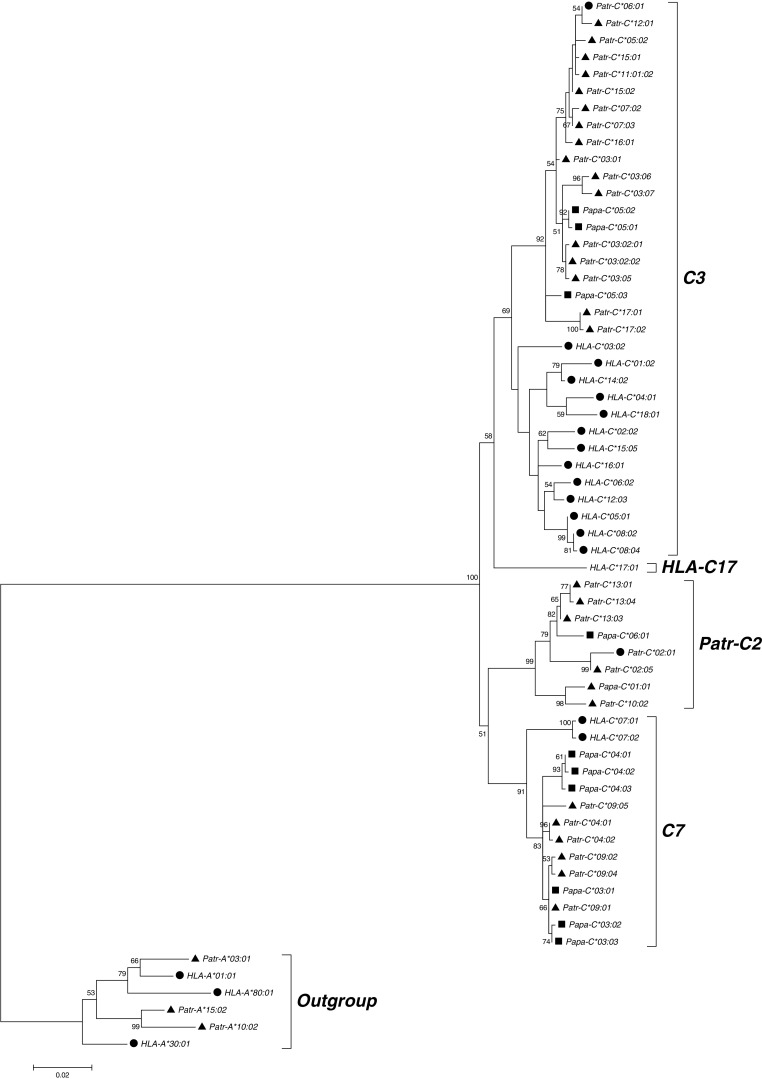
Molecular phylogenetic tree of MHC class I *C* sequences reconstructed using the maximum likelihood method based on the Tamura 3-parameter model. The model used a discrete gamma distribution allowing evolutionary rate differences among sites. The tree with the highest log likelihood (−4873.9977) is shown. Confidence in the branching patterns is indicated by bootstrap values and only relevant values (≥50) are shown. The scale indicates the number of substitutions per site. The tree was rooted with an outgroup consisting of the indicated *MHC-A* sequences. The three species humans, chimpanzees, and bonobos are represented by *circles*, *triangles*, and *squares*, respectively, in front of the sequence names. Different phylogenetic groups are defined on the right side of the tree, showing the two lineages *C3* and *C7* and as well the species specific lineages *HLA-C17* and *Patr-C2*

To examine the evolutionary relationships of the alleles from the two *Pan* species, we reconstructed phylogenetic trees from the coding region sequences for the *A*, *B*, and *C* loci. We included chimpanzee and bonobo coding region sequences from the literature to depict all available species diversity and all known phylogenetic groups. We found that for the *A* locus, human alleles clustered into two lineages *A2* and *A3*, as previously described (Lawlor et al. 1990) (Fig. 1). As expected, chimpanzee and bonobo sequences clustered only within the human *A3* lineage of the *A* locus. Furthermore, chimpanzee alleles formed two groups of moderate bootstrap support previously termed *Patr-A1* and *Patr-A4* (Adams et al. 2000), with bonobo alleles found in both chimpanzee groups. Phylogenetic analysis of the alleles from the *B* locus produced a tree with notably low bootstrap support (Fig. 2). Nonetheless, it is notable that in contrast to bonobos, chimpanzee alleles were found in more phylogenetic clades, hinting at a greater diversity in this species at this locus. The phylogenetic tree for the *C* locus (Fig. 3) featured four different lineages named *C3*, *C7*, *HLA-C17*, and *Patr-C2* with intermediate bootstrap support (Adams et al. 2000). As was previously described, both chimpanzee and human alleles are found in the lineages *C3* and *C7*, whereas each species also has an additional lineage where sequences from the other species are not found (*Patr-C2* and *HLA-C17*) (Adams et al. 2000). Bonobo alleles clustered together with chimpanzee alleles within the *C3*, *C7*, and *Patr-C2* lineages. However, chimpanzees seem to have much more variety within the *C3* lineage, suggesting again relatively lower diversity in bonobos.

After seeing that bonobos have relatively fewer alleles than chimpanzees and that the bonobo alleles appear to be less widespread in the phylogenetic trees, we next estimated nucleotide diversities of all exons to quantitatively assess whether bonobos have a less diverse set of *A*, *B*, and *C* alleles than chimpanzees. Because chimpanzees as a species exhibit more genetic structure than bonobos (Becquet et al. 2007; Kawamoto et al. 2013; Prado-Martinez et al. 2013), we compared nucleotide diversity estimates from our bonobo samples with those from our central chimpanzee samples as well as with published western chimpanzees (Adams et al. 2000) and humans (Yoruba) (The International HapMap C 2005).

**Fig. 4:**
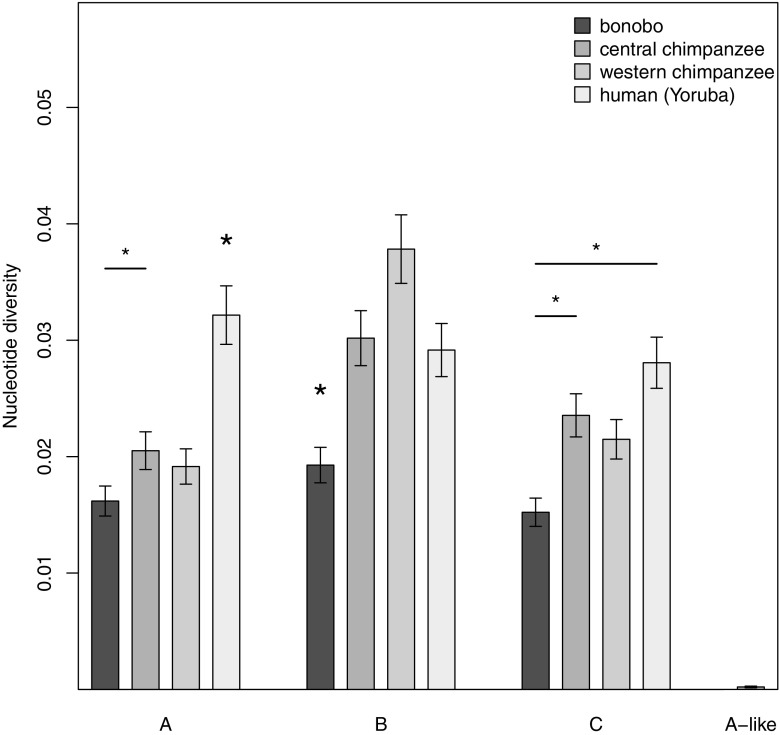
Estimated nucleotide diversity (∏) of all exons for the different MHC loci *A*, *B*, *C*, and *A-like* DNA sequences from 20 unrelated bonobos and central chimpanzees reported here and 20 western chimpanzees and 20 humans (Yoruba) from the literature (Adams et al. 2000; The International HapMap C 2005). *Error bars* represent the standard error of the mean. Significant differences are indicated by *stars* above the individual bars, whereby *small stars* indicate significant differences between two bars linked by a line and *big stars* indicate significant differences to every other bar at this particular locus (permutation test, 10,000 resamplings, Bonferroni correction, *p* ≤ 0.002)

Our dataset of 20 bonobos exhibited significantly lower nucleotide diversity than our dataset of 20 central chimpanzees and humans at all three loci (Fig. 4 and supplement tables S3 and S4 for the exact values), while bonobos and western chimpanzees had no significant differences in diversity estimates at the *A* and *C* loci. At the *A* locus, our sample of humans had significantly higher nucleotide diversity than any of the *Pan* taxa. At the *B* locus, however, our sample of 20 bonobos had less nucleotide diversity than any of the other taxa. Compared to the other three loci *A*, *B*, and *C*, central chimpanzees had extremely low nucleotide diversity at the *A-like* locus.

**Fig. 5:**
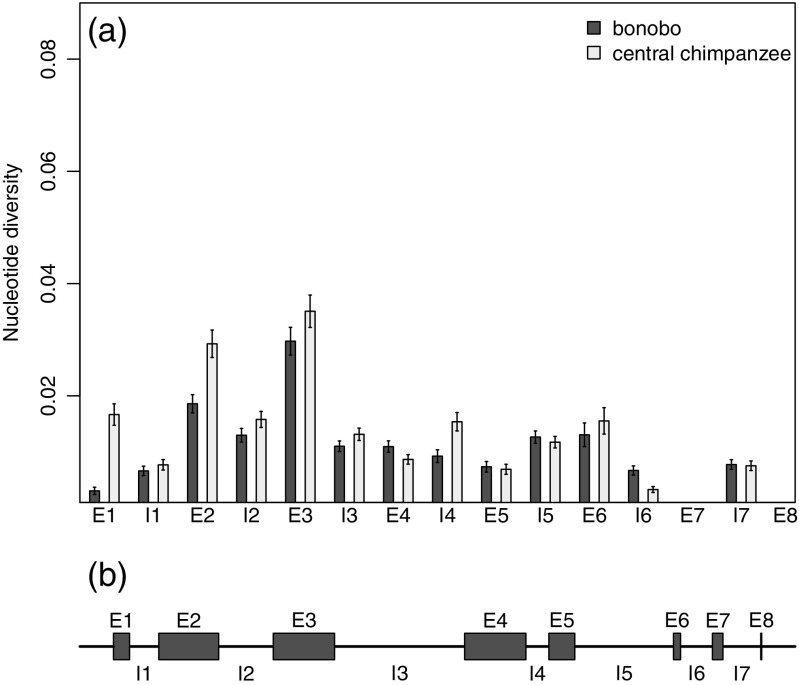
**a** Estimated nucleotide diversity (∏) at the class I *A* locus for each exon (E1–E8) and intron (I1–I7) for 20 bonobos and 20 central chimpanzees. *Error bars* represent standard error of the mean. **b** Organization of exons and introns of the *MHC-A* gene. The length of the boxes indicates the relative length of the particular exons and introns, where I3 is 583 bp long and E8 is 5 bp long

**Fig. 6:**
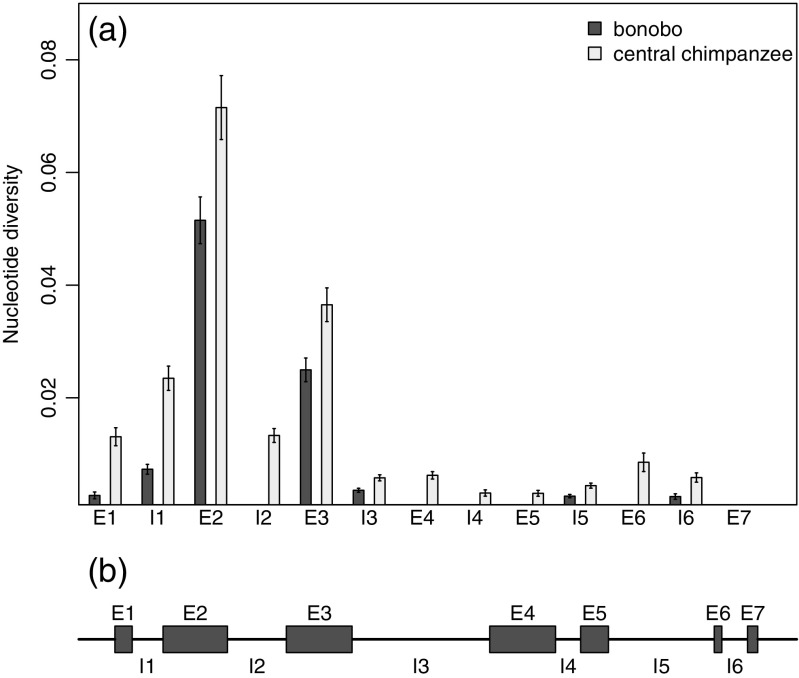
**a** Estimated nucleotide diversity (∏) of the class I *B* locus for each exon (E1–E7) and intron (I1–I6) for 20 bonobos and 20 central chimpanzees. *Error bars* represent standard error of the mean. **b** Organization of exons and introns of the *MHC-B* gene. The length of the boxes indicates the relative length of the particular exons and introns, where I3 is 574 bp long and E7 is 44 bp long

**Fig. 7:**
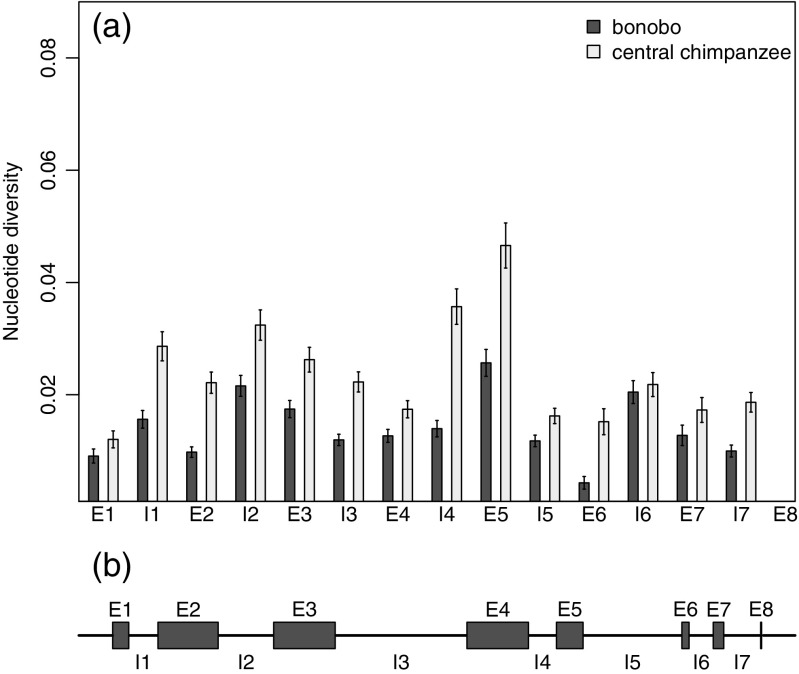
**a** Estimated nucleotide diversity (∏) of the class I *C* locus for each exon (E1–E8) and intron (I1–I7) for 20 bonobos and 20 central chimpanzees. *Error bars* represent standard error of the mean. **b** Organization of exons and introns of the *MHC-C* gene. The length of the boxes indicates the relative length of the particular exons and introns, where I3 is 588 bp long and E8 is 5 bp long

To understand the patterns of relative nucleotide diversity across the MHC class I *A*, *B*, and *C* genes, we used our sequences of the entire genes from bonobos and central chimpanzees to estimate nucleotide diversity for each individual exon and intron (Figs. 5, 6, and 7). We found that there was no particular exon or intron within the genes which could explain the lower nucleotide diversity of bonobos. It was rather a general reduced diversity, e.g., lower diversity across all exons and introns. The comparison between the three different genes reveals that the distribution of nucleotide diversity of the different exons and introns was not similar between the three loci. For both species, the nucleotide diversity for the *A* locus (Fig. 5) was highest in exon 2 and exon 3, followed by lower nucleotide diversity for other regions of the gene. The nucleotide diversity of the *B* locus (Fig. 6) follows the same pattern, but with greater differences between the highly diverse exons 2 and 3 and the other regions of the gene. Nucleotide diversity of those other regions in bonobos was occasionally zero (intron 2, exon 4, intron 4, exon 6, exon 6, exon 7), which means that all bonobo *B* alleles within our sample set share the same sequence in those parts of the gene. The pattern of nucleotide diversity for the *C* locus differed from the other two loci (Fig. 7). At the *C* locus, the highest nucleotide diversity for both species was within exon 5. The nucleotide diversity of exon 2 and exon 3, which had the highest diversity values in the *A* and *B* loci, had relatively low nucleotide diversity compared to other parts of the gene.

## Discussion

This study of MHC class I diversity in the chimpanzee and bonobos identified 43, 54, 40, and 11 full-length alleles at the *A*, *B*, *C*, and *A-like* loci. Of these, 19, 23, 20, and 1 defined coding region sequences had not been previously described, thereby emphasizing the extraordinary polymorphism at this gene complex and the utility of investigating additional individuals to fully describe the whole range of MHC sequences in the genus *Pan*. In particular, the new sequences from our study doubles the number of known bonobo MHC class I alleles for each of the three loci *A*, *B*, and *C*.

The lower nucleotide diversity at the *A* locus of chimpanzees and bonobos compared to humans is not surprising due to the absence of one of the two lineages at the *A* locus in those two species (Adams et al. 2000; Lawlor et al. 1995; McAdam et al. 1995). Human *A* alleles cluster in the two ancient lineages *A2* and *A3* where chimpanzee and bonobo *A* alleles are only related to alleles of the *A3* linage (Lawlor et al. 1990; McAdam et al. 1995). It has been suggested by de Groot et al. (2002) that the loss of the *A2* lineage in chimpanzees was due to a selective sweep, perhaps caused by a widespread simian immunodeficiency virus (SIV) infection. Under this assumption, alleles from the *A2* lineage offered insufficient protection against this infection and were therefore lost during evolution (Gleimer et al. 2011). The fact that bonobos also contain sequences only related to the *A3* lineage indicates that if the selective sweep happened, it occurred before the separation of the two species. However, despite the loss of this specific *A* lineage, the *Patr-A-like* gene in chimpanzees has an overlapping peptide binding repertoire to human alleles from the *A2* lineage, giving chimpanzees with this locus the ability to bind peptides specific to the A02 supertype (Gleimer et al. 2011). This is not possible in bonobos as they lack the *A-like* locus, and whether the immune response of bonobos is affected by this or if bonobos compensate this lineage loss in a different way requires further investigation.

In comparison to central chimpanzees, our results indicate that bonobos have a decreased MHC class I diversity shown by the lower number of different alleles, the phylogenetic trees and the decreased nucleotide diversity at all three loci. However, this lower diversity was larger at the *B* and *C* loci compared to *A* locus. One possible explanation for this reduced diversity in bonobos is that it simply reflects an overall reduced genetic diversity in bonobos. Sequencing of different non-coding autosomal regions distributed over different parts of the genome as well as sequencing of the whole genome for several individuals revealed reduced nucleotide diversity and a lower effective population size in bonobos compared to central chimpanzees (Fischer et al. 2006; Fischer et al. 2011; Prado-Martinez et al. 2013). However, western chimpanzees and humans also exhibit low diversity and effective population size (Li and Sadler 1991; Nam et al. 2017; Takahata et al. 1995; Zhao et al. 2000). If the MHC diversity reflects only the overall genetic diversity of the species, we would expect also a reduced MHC diversity in western chimpanzees and humans as compared to central chimpanzees, but our results show that published western chimpanzee and human sequences have equal or higher MHC nucleotide diversity than central chimpanzee sequences. This suggests that the low MHC diversity in bonobos is unlikely to reflect only the overall low genetic diversity of this species.

The critical role of the MHC in relation to immune functions rather suggests that the reduced bonobo MHC diversity may be explained by a selective process caused by pathogen exposure in the past history of bonobos since their spilt from chimpanzees. In such a scenario, exposure to certain pathogens would change the frequencies of certain MHC alleles within the population and may lead to a loss of certain MHC alleles and lineages. This loss could occur due to following scenario: in a selective sweep, certain MHC alleles give protection against certain parasites or pathogens and are therefore selected within the population leading to an increase in frequency while disadvantageous MHC alleles at this locus are lost via purifying selection. Because we found the greatest difference in diversity at the *B* locus, this process of selection could have affected mainly this locus and due to genetic hitchhiking also the closely located *C* locus (Anzai et al. 2003; Shiina et al. 2006). Evidence for the role of MHC class I alleles in increased protection or susceptibility against diseases have been observed in humans. Human alleles HLA-B*57, B*27, or B*53 are associated with an increased protection against AIDS or malaria, respectively, whereas the allele HLA-B*35 was linked to rapid progression to AIDS after HIV infection (Altfeld et al. 2003, 2006; Carrington et al. 1999; Feeney et al. 2004; Gao et al. 2001; Hill et al. 1991; Hill et al. 1992; Kaslow et al. 1996; Migueles et al. 2000). It remains to be investigated which pathogens could be responsible for the loss in MHC diversity in bonobos. Potential candidates could be pathogens which also have a critical impact on health conditions in the two closely related species chimpanzees and humans like malaria or SIV/HIV. Although identical methods have been used to screen bonobo and chimpanzee samples from the wild, SIV has not been detected in bonobos thus far (Li et al. 2012; Sharp and Hahn 2011). The apparent absence of SIV in bonobos could be explained by the assumption that this species never experienced SIV or exhibited a superior defense against this type of virus similar as proposed for western chimpanzees (de Groot et al. 2002). Other studies showed that bonobos are confronted with malaria, ebola, monkeypox, and trypanosomiasis in their natural habitat and that pathogens have an impact on their distribution (Inogwabini and Leader-Williams 2012; Krief et al. 2010). One of those pathogens could also have been important in shaping the bonobo MHC diversity in their evolutionary past but this remains speculative at this point.

In concordance with our results, a recently published study by Wroblewski et al. (2017) investigated sequences of exons 2 and 3 of MHC class I *B* in representatives of several wild bonobo populations and found low diversity. Interestingly, the allele with the highest frequency (Papa-B*07:01) in their study was also the most common bonobo *B* allele found in our study (Table 2).

Our study demonstrates the utility of typing MHC alleles using PCR and the PacBio RS II sequencing technology in set of wild-born individuals. The comparison between chimpanzees and bonobos revealed a lower MHC diversity in bonobos, representing perhaps the result of a selective process after the split from chimpanzees. Investigating also the effect of this low MHC diversity on the parasite and pathogen resistance in this species could contribute to our understanding of the relationship between MHC diversity and immunity.

## Electronic supplementary material

ESM 1 (DOCX 28 kb)
