# Supplementary material for: MHC class I diversity in chimpanzees and bonobos
Source: Immunogenetics. 2017 Jun 16;69(10):661–76. doi: 10.1007/s00251-017-0990-x (PMC5597694; doi:10.1007/s00251-017-0990-x)
Supplement: Supplementary file 1 — (DOCX 28 kb) [file 251_2017_990_MOESM1_ESM.docx]

**Supplementary Material** MHC class I diversity in chimpanzees and bonobos

Table S1 sample details, DRC - Democratic Republic Congo

| no. | name | species | subspecies | geographical location | wild born | sex |
| --- | --- | --- | --- | --- | --- | --- |
| 1 | Ulindi | Pan paniscus |  |  | no | f |
| 2 | Likasi | Pan paniscus |  | DRC | yes | f |
| 3 | Malou_L | Pan paniscus |  | DRC | yes | f |
| 4 | Lodja | Pan paniscus |  | DRC | yes | f |
| 5 | Bandundu | Pan paniscus |  | DRC | yes | f |
| 6 | Boende | Pan paniscus |  | DRC | yes | m |
| 7 | Keza | Pan paniscus |  | DRC | yes | m |
| 8 | Kikwit | Pan paniscus |  | DRC | yes | m |
| 9 | Kisantu | Pan paniscus |  | DRC | yes | f |
| 10 | Kubulu | Pan paniscus |  | DRC | yes | m |
| 11 | Lipopo | Pan paniscus |  | DRC | yes | m |
| 12 | Lomami | Pan paniscus |  | DRC | yes | m |
| 13 | Max | Pan paniscus |  | DRC | yes | m |
| 14 | Semendwa | Pan paniscus |  | DRC | yes | f |
| 15 | Tshilomba | Pan paniscus |  | DRC | yes | f |
| 16 | Matadi | Pan paniscus |  | DRC | yes | m |
| 17 | Isiro | Pan paniscus |  | DRC | yes | f |
| 18 | Bili_L | Pan paniscus |  | DRC | yes | m |
| 19 | Bolobo | Pan paniscus |  | DRC | yes | m |
| 20 | Fizi | Pan paniscus |  | DRC | yes | m |
| 21 | Api | Pan paniscus |  | DRC | yes | m |
| 22 | Fan Tuek | Pan troglodytes | troglodytes | Le Congo; Zoo Brazza | yes | f |
| 23 | Marcelle | Pan troglodytes | troglodytes |  | yes | f |
| 24 | Bayokele | Pan troglodytes | troglodytes | Le Congo; Brazza | yes | f |
| 25 | Clara_T | Pan troglodytes | troglodytes | Le Congo; Zoo PNR | yes | f |
| 26 | Bimangou | Pan troglodytes | troglodytes | Le Congo; Zoo PNR | yes | m |
| 27 | Botsomi | Pan troglodytes | troglodytes | Le Congo | yes | f |
| 28 | Casimir | Pan troglodytes | troglodytes | DRC | yes | m |
| 29 | Golfi | Pan troglodytes | troglodytes | Le Congo; Zoo Brazza | yes | f |
| 30 | Grand Maitre | Pan troglodytes | troglodytes | Le Congo; Loukoulela | yes | m |
| 31 | Imphondo | Pan troglodytes | troglodytes | Le Congo; Imphondo | yes | f |
| 32 | Loufoumbou | Pan troglodytes | troglodytes |  | yes | m |
| 33 | Lufino | Pan troglodytes | troglodytes |  | yes | m |
| 34 | Moka | Pan troglodytes | troglodytes | Le Congo; Kakamoueka | yes | m |
| 35 | Castro | Pan troglodytes | troglodytes |  | yes | m |
| 36 | Bailele | Pan troglodytes | troglodytes |  | yes | m |
| 37 | Elikia | Pan troglodytes | troglodytes | Le Congo; | yes | m |
| 38 | Agnagui | Pan troglodytes | troglodytes | Le Congo; Loukolela | yes | f |
| 39 | Dzeke | Pan troglodytes | troglodytes | Le Congo; Impfondo | yes | m |
| 40 | Gao | Pan troglodytes | troglodytes | Le Congo; PNR | yes | f |
| 41 | Chinoc | Pan troglodytes | troglodytes | Le Congo | yes | m |
| 42 | Diana | Pan troglodytes | schweinfurthii | DRC-South | yes | f |
| 43 | Cleo | Pan troglodytes | schweinfurthii | Zambia | yes | f |
| 44 | Bihati | Pan troglodytes | schweinfurthii | DRC-Kinshasa | yes | f |
| 45 | Trixie | Pan troglodytes | schweinfurthii | DRC-Rwanda | yes | f |
| 46 | Maya | Pan troglodytes | schweinfurthii | DRC-Mbuji Mayi | yes | f |
| 47 | Tongo | Pan troglodytes | schweinfurthii | Rwanda | yes | m |
| 48 | Cindy | Pan troglodytes | verus | Ivory coast | yes | f |
| 49 | Berta | Pan troglodytes | verus | Ivory coast | yes | f |
| 50 | Linda | Pan troglodytes | verus | Liberia | yes | f |
| 51 | Alice | Pan troglodytes | verus | Ivory coast | yes | f |

Table S2 Primer sequences

| Locus | Name | Sequence 5’- 3’ |
| --- | --- | --- |
| A | HCG-X_enhA_fwd | CGGTGTATGGATTGRGGAGKCCCAG |
|  | HCG-A_3'UTR_rev | GAAGGAABAAGTTACAGCTCAGTGCACC |
| B | *HCG_enhB_fwd** | GTCGKGTCCTTCTTCCRGGATACTCG |
|  | *HCG-B_3'UTR_rev** | GCCAGCTGTCTCAGGCTACAGAAAACAAC |
| C | *HCG_enhC_fwd** | CGWCGGGTCCTTCTTCCTGAATACTCA |
|  | *HCG_C_3'UTR_rev** | TGCATCTCAGTCCCACACAGGCAG |
| A-like | Patr-Al_full_fwd | CAACCTGCGTCGGTTCCTTCTTCCTAG |
|  | Patr-Al_full_rev | ACACATGCAGGTGCCTTTGCAGAAAGTA |

*Primer sequences were taken from Hans et al. (2017)

Table S3 Nucleotide diversity estimates

| species/subspecies | Bonobo | | | central Chimpanzee | | | | western chimpanzee | | | human (Yoruba) | | |
| --- | --- | --- | --- | --- | --- | --- | --- | --- | --- | --- | --- | --- | --- |
| locus | A | B | C | A | B | C | A-like | A | B | C | A | B | C |
| nucleotide diversity ∏ | 0.0162 | 0.0193 | 0.0152 | 0.0205 | 0.0309 | 0.0235 | 0.0002 | 0.0192 | 0.0378 | 0.0215 | 0.0322 | 0.0292 | 0.0281 |
| standard deviation of ∏ | 0.0082 | 0.0096 | 0.0077 | 0.0102 | 0.0153 | 0.0117 | 0.0003 | 0.0096 | 0.0186 | 0.0107 | 0.0159 | 0.0144 | 0.0139 |
| standard error of mean | 0.0013 | 0.0015 | 0.0012 | 0.0016 | 0.0024 | 0.0019 | 0.0001 | 0.0015 | 0.0029 | 0.0017 | 0.0025 | 0.0023 | 0.0022 |

Table S4 P-values of nucleotide diversity estimates. P-values were adjusted for multiple testing using Bonferroni correction (p ≤ 0.002). Values in bold are considered as being significant.

| **A Locus** | bonobo | central chimpanzee | western chimpanzee | human (Yoruba) |
| --- | --- | --- | --- | --- |
| bonobo | - | - | - | - |
| central chimpanzee | **0.0009** | - | - | - |
| western chimpanzee | 0.091 | 0.4281 | - | - |
| human (Yoruba) | **0.0001** | **0.0001** | **0.0003** | - |
|  |  |  |  |  |
| **B Locus** | bonobo | central chimpanzee | western chimpanzee | human (Yoruba) |
| bonobo | - | - | - | - |
| central chimpanzee | **0.0001** | - | - | - |
| western chimpanzee | **0.0001** | 0.0086 | - | - |
| human (Yoruba) | **0.0001** | 0.5766 | 0.0028 | - |
|  |  |  |  |  |
| **C Locus** | bonobo | central chimpanzee | western chimpanzee | human (Yoruba) |
| bonobo | - | - | - | - |
| central chimpanzee | **0.0013** | - | - | - |
| western chimpanzee | 0.054 | 0.4447 | - | - |
| human (Yoruba) | **0.0001** | 0.0122 | 0.0042 | - |

Literature

Hans JB, Bergl RA, Vigilant L (2017) Gorilla MHC class I gene and sequence variation in a comparative context Immunogenetics:1-21 doi:10.1007/s00251-017-0974-x
